# Effects of high-intensity interval training (HIIT) versus moderate-intensity continuous training (MICT) on cardiopulmonary function, body composition, and physical function in cancer survivors: a meta-analysis of randomized controlled trials

**DOI:** 10.3389/fphys.2025.1594574

**Published:** 2025-06-13

**Authors:** Chenggen Peng, Ming Hu, Linlin Yang, Zhichao Yuan

**Affiliations:** ^1^ Hunan Agricultural University, Changsha, Hunan, China; ^2^ Faculty of Sport and Health Sciences, Guangxi Science & Technology Normal University, Laibin, Guangxi, China; ^3^ College of Sports, Hunan International Economics University, Changsha, Hunan, China; ^4^ School of Sports Science, Changsha Normal University, Changsha, Hunan, China

**Keywords:** HIIT, MICT, cancer survivors, cardiopulmonary function, body composition, physical function, meta-analysis

## Abstract

**Background:**

Advances in cancer treatment have led to a significant increase in the global number of cancer survivors. However, long-term health management challenges—such as reduced cardiopulmonary function, cancer-related fatigue, and metabolic dysregulation—remain formidable. The purpose of this study was to conduct a meta-analysis of randomized controlled trials (RCTs) to comprehensively compare the effects of high-intensity interval training (HIIT) versus moderate-intensity continuous training (MICT) on Cardiopulmonary function, body composition, and physical function in cancer survivors. Thereby providing evidence-based guidance for individualized exercise prescriptions.

**Methods:**

By the PRISMA guidelines, we systematically searched databases including PubMed, Web of Science, Scopus, Embase, the Cochrane Library, and EBSCOhost up to February 2025. A total of 12 eligible RCTs were included, breast cancer (n = 7), colorectal cancer (n = 3), and mixed cancer types (n = 2). Meta-analysis was performed using Cochrane Collaboration’s Review Manager 5.4, while sensitivity analyses were conducted with Stata MP 14.0 to assess the stability and reliability of the results. Egger’s test was utilized to evaluate the presence of publication bias.

**Results:**

The meta-analysis revealed that, compared with MICT, HIIT was significantly more effective in improving VO_2_ peak (Peak Oxygen Uptake) in cancer survivors [SMD = 0.53, 95% CI (0.21, 0.84), Z = 3.30, P = 0.001]. However, no statistically significant differences were found between HIIT and MICT in terms of body composition (including Body Mass, Total Fat Mass, Lean Body Mass, Fat Percentage, Body Mass Index, Waist Circumference, and Hip Circumference) or physical function (including Sit-to-Stand Test and 6-Minute Walk Test).

**Conclusion:**

HIIT appears superior to MICT in enhancing VO_2_ peak and, consequently, cardiopulmonary function in cancer survivors. Nonetheless, both training modalities yield comparable outcomes in body composition and physical function. Given the variability in the quantity and quality of the included studies, further well-designed and objective RCTs are warranted to validate these findings.

**Systematic Review registration:**

https://www.crd.york.ac.uk/PROSPERO/myprospero, identifier CRD420250654968.

## 1 Introduction

Cancer is a group of diseases characterized by abnormal cell growth. It has become a major social, public health, and economic issue in the 21st century, and is one of the deadliest diseases worldwide, claiming millions of lives each year (Matthews et al., 2022). Statistics indicate that approximately one in five men or women will develop cancer during their lifetime, with about one in nine men and one in every twelve women succumbing to the disease (Bray et al., 2024; Xiong et al., 2024). However, with advances in early cancer screening and treatment techniques, the global number of cancer survivors has increased significantly (Costa et al., 2020; He et al., 2024). Cancer survivors are defined as individuals who survive during or after cancer treatment (Denlinger and Barsevick, 2009; Denlinger et al., 2014). Although the number of cancer survivors continues to rise and the quality of care has improved markedly, these individuals may still experience severe complications, cancer recurrence-associated mortality, and treatment-related adverse events (Chen et al., 2024). The primary treatment modalities for cancer survivors include surgery, chemotherapy, radiotherapy, and immunotherapy. However, these treatments are often accompanied by severe side effects, such as reduced cardiopulmonary function, muscle atrophy, fatigue syndrome, and psychological disorders, which significantly impair the long-term quality of life (Jereczek-Fossa et al., 2002; von Kemp and Cosyns, 2023).

Conventional cancer treatments mainly cover radiotherapy, surgery, and chemotherapy. Although a wide range of chemotherapeutic agents exist, their effectiveness is limited by several factors (Xu et al., 2023; Xu et al., 2024). Therefore, Mokashi et al. researched and explored novel phytonutrients and herbal materials as potential treatments based on Ayurvedic medicine (Mokashi and Bhatia, 2024). Zhang developed an image recognition program using AI techniques that was able to extract implicit information from a human face and effectively differentiate between a cancer patient and a healthy individual. The study revealed that the physiological basis of the AI observer lies in the close link between craniofacial genes and cancer susceptibility genes (Zhang et al., 2022). Guo et al. proposed that physical stimulation modulates the tumor microenvironment by altering the tumor vasculature system, remodeling the extracellular matrix, and activating the immune response, to achieve the goal of adjuvant to other tumor therapies (Guo et al., 2024). Additionally, a study by Luo et al. noted that leisure-time physical activity, defined as non-specific physical activity with an intensity of three or more metabolic equivalents across a range of activities, significantly reduces cancer risk and delays progression in patients (Luo et al., 2024).

Some studies have indicated that exercise, as a non-pharmacological intervention, has profound effects on improving chronic diseases related to metabolic syndrome, cardiovascular and pulmonary disorders, musculoskeletal and joint conditions, as well as cancer (Pedersen and Saltin, 2006; Mitchell and Barlow, 2011). Moreover, exercise interventions have been shown to effectively enhance the physiological function of cancer survivors, alleviate treatment-related toxicities, and reduce the risk of recurrence (Daum et al., 2016; Furmaniak et al., 2016; Schmitt et al., 2016; Cramer et al., 2017; Mustian et al., 2017; Hilfiker et al., 2018; Nakano et al., 2018; Yang et al., 2020) Previous research has demonstrated that physical activity can significantly increase peak oxygen uptake (VO_2_ peak) in cancer survivors (Lee et al., 2019; Foulkes et al., 2023).

For most cancer survivors, lack of time and difficulties in maintaining regular exercise are common barriers to participation (Elshahat et al., 2021; Cariolou et al., 2023). High-intensity interval training (HIIT), as a structured and enhanced form of interval training involving short bouts of high-intensity exercise, is a time efficient approach (Weston et al., 2014; Batacan et al., 2017). HIIT fits the current situation of cancer survivors’ lack of time due to its time efficiency, which may be one of the reasons for its high adherence, e.g., Isanejad et al. reported that exercise adherence in the HIIT group was superior to that of the MICT group (participation rate: 98% vs. 92%) (Isanejad et al., 2023). Dolan et al. showed that the adherence rate in the HIIT group was 100% compared to approximately 91.67% in the MICT group (Dolan et al., 2016). Additionally, Devin et al. noted that the intervention completion rates for the HIIT and MICT groups were 94.7% and 89.5%, respectively (Devin et al., 2018). In addition, studies have shown that HIIT is more enjoyable than low or moderate-intensity aerobic exercise, and this enjoyment enhances participants’ motivation to exercise, which in turn has a positive impact on compliance (Bartlett et al., 2011; Gillen and Gibala, 2014; Thum et al., 2017). Bartlett et al. showed higher ratings of post-exercise perceived enjoyment after interval running compared to continuous running (p < 0.05) (Bartlett et al., 2011). Although some studies have shown higher adherence to HIIT, the results of these studies may be confounded by several factors, such as the study by Hooshmand Moghadam et al. which showed that the adherence rate in both the HIIT group and the MICT group was approximately 86.67% (Hooshmand Moghadam et al., 2021). Therefore, the results of the study need to be interpreted with caution as exercise adherence in cancer patients is influenced by multidimensional factors such as exercise intensity, psychological support, and individual health status.

Some evidence suggests that HIIT is associated with more pronounced improvements in VO_2_ peak and certain metabolic parameters while requiring less time compared to MICT (Gibala et al., 2014). However, other studies have indicated that HIIT does not result in greater enhancements in VO_2_ peak than MICT (Bell et al., 2021). The differences between HIIT and MICT in terms of cardiopulmonary health and vascular function appear to be minimal (Weston et al., 2014; Milanović et al., 2015; Ramos et al., 2015). One meta-analysis found no significant difference in VO_2_ peak between HIIT (n = 56) and MICT (n = 43) (p = 0.15) (Wallen et al., 2020). Moreover, there is a paucity of meta-analyses comparing the effects of HIIT and MICT in cancer survivors. Therefore, the primary objective of this systematic review and meta-analysis was to evaluate the effects of HIIT versus MICT in cancer survivors, with VO_2_ peak as the primary outcome and body composition and physical function as secondary outcomes.

## 2 Methods

This systematic review was registered with the International Prospective Register of Systematic Reviews (registration number: CRD420250654968). This systematic review and meta-analysis were conducted by the Preferred Reporting Items for Systematic Reviews and Meta-Analyses (PRISMA 2020) guidelines.

### 2.1 Search strategy

A comprehensive search was conducted in databases including PubMed, Web of Science, Scopus, Embase, the Cochrane Library, and EBSCOhost, covering the period from their inception to February 2025. The search was limited to articles published in English. Reference management was performed using EndNote (version X9). In addition to investigating the effects of HIIT and MICT on cancer survivors, reference tracking was also conducted for previously published trials and meta-analyses in this field.

The search strategy involved the use of Medical Subject Headings (MeSH terms), including “Cancer Survivors,” “High-Intensity Interval Training,” and “Randomized Controlled Trial,” as well as their free-text equivalents. Non-MeSH terms such as “Moderate-Intensity Continuous Training” and their synonyms were also employed, combined using Boolean operators. The article search was primarily conducted by two authors, HM and YLL. In cases of disagreement, a third author, PCG, was consulted to reach a consensus. All analyses were based on previously published studies; therefore, neither ethical approval nor informed consent from patients was required (Supplementary Table S1).

### 2.2 Inclusion and exclusion criteria

#### 2.2.1 Inclusion criteria

(1) Language: Studies published in English only.
(2) Participants: Adult cancer survivors.
(3) HIIT Group: Supervised or unsupervised HIIT (clearly defined high-intensity interval protocols, such as 80%–95% of maximum heart rate).
(4) MICT Group: Supervised or unsupervised MICT (moderate-intensity continuous training, such as 50%–70% of maximum heart rate).
(5) Outcomes: Cardiovascular function, body composition, and physical function.
(6) Study Design: Randomized controlled trials (RCTs).

#### 2.2.2 Exclusion criteria

(1) Non-randomized studies, animal experiments, or studies with mixed interventions that did not distinguish between HIIT and MICT.
(2) Studies lacking quantitative results or relevant outcome measurements.
(3) Studies with incomplete data or those for which the full text was unavailable.
(4) Studies involving multimodal exercise interventions (such as HIIT combined with resistance training).

### 2.3 Data extraction

In the initial search, all references were imported into EndNote (version X9). After removing duplicates, studies unrelated to the topic were excluded based on their titles. Further screening was conducted by reviewing abstracts and full texts. The extracted information included basic details (author, publication year, country, cancer type, gender, age, and sample size) and study characteristics (type of intervention, intervention frequency, intervention measures, duration, intensity, and outcome measures). For all groups, the mean changes from baseline and the standard deviations (SDs) of these changes, as well as the number of participants at each assessment, were extracted. In case of missing ending data, priority was given to obtaining the original data by contacting the original authors, and if this was not possible, reasonable extrapolations were made from the available data. If there was a situation where key data were missing altogether and the data could not be obtained, the study was deleted. If variables were reported at multiple time points during the intervention, only pre- and post-intervention time points were included. The primary outcome was VO_2_peak, and the secondary outcomes were body composition and physical function. Data extraction and summary were performed by two authors (HM and YLL). In cases of disagreement, a third author (PCG) was consulted to reach a decision.

### 2.4 Risk of bias

The risk of bias for the included RCTs was assessed using the Cochrane Collaboration’s Risk of Bias 2 tool (RoB 2; version 2). This tool evaluates potential bias across five domains: the randomization process, deviations from intended interventions, missing outcome data, measurement of outcomes, and selection of the reported results. A study was judged to have a “low risk of bias” if all domains were rated as low risk. If at least one domain was rated as “some concerns,” the study was judged to have “some concerns.” A study was considered to have a “high risk of bias” if at least one domain was rated as high risk or if ≥3 domains were rated as “some concerns.” The risk of bias was independently assessed by two researchers (PCG and YLL). Any disagreements were resolved through consensus or, if necessary, by consulting a third researcher (HM).

### 2.5 Data analysis

Meta-analysis was conducted using Review Manager 5.4 software provided by the Cochrane Collaboration. Statistical significance was defined as a p-value <0.05. Since all outcomes were continuous variables, the standardized mean difference (SMD) and 95% confidence intervals (95% CI) were calculated. Heterogeneity was assessed using the I^2^ statistic. An I^2^ > 50% was considered to indicate significant heterogeneity. A random-effects model was used when I^2^ ≥ 50%, and a fixed-effects model was applied when I^2^ < 50%. If heterogeneity was substantial (I^2^ ≥ 50%), subgroup analysis or sensitivity analysis was performed to interpret the results. Differences between groups were considered not significant if the SMD overlapped with zero. Stata MP 14.0 software was used for sensitivity analysis to determine the stability and reliability of the results. Egger’s test was employed to assess potential publication bias.

## 3 Results

### 3.1 Search outcomes

**FIGURE 1:**
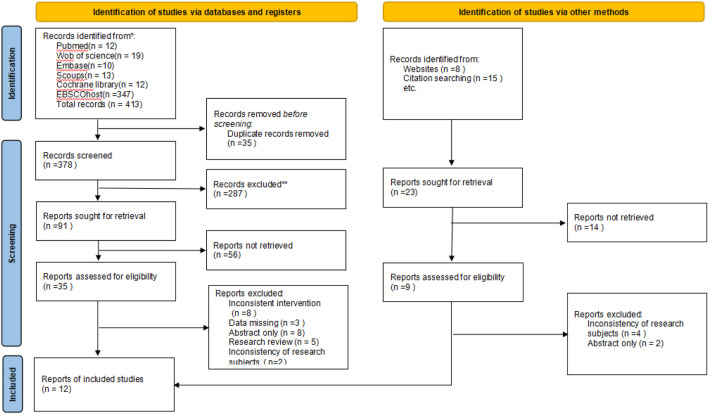
PRISMA flowchart.

The initial search yielded 413 articles, including 12 from PubMed, 19 from Web of Science, 13 from Scopus, 10 from Embase, 12 from Cochrane Library, and 347 from EBSCOhost. After removing 35 duplicate studies using EndNote, 287 articles were excluded based on their titles. According to the inclusion and exclusion criteria, 56 articles were removed after the abstract screening. Full-text review led to the exclusion of 26 studies, including 8 due to inconsistent interventions, 3 for missing data, 8 that were abstract-only, 5 research reviews, and 2 due to inconsistencies in research subjects. Additionally, 23 articles were identified through website and citation searching. After reviewing and excluding duplicates and non-relevant studies, 3 articles were included in the meta-analysis. Ultimately, 12 studies met the eligibility criteria and were included in the analysis (Figure 1).

### 3.2 Characteristics of the studies

From 2015 to 2023, a total of 12 studies were included in the meta-analysis: 1 from New Zealand (Bell et al., 2021), 6 from Australia (Devin et al., 2016; Toohey et al., 2016; Devin et al., 2018; Toohey et al., 2018; Northey et al., 2019; Toohey et al., 2020), 1 from Canada (Dolan et al., 2016), 2 from Iran (Hooshmand Moghadam et al., 2021; Isanejad et al., 2023), 1 from the United States (Moraitis et al., 2023), and 1 from Germany (Schmitt et al., 2016). The sample sizes ranged from 7 to 47 participants, with ages varying between 39 and 75 years. Based on cancer types, 7 studies focused on breast cancer survivors (Dolan et al., 2016; Schmitt et al., 2016; Northey et al., 2019; Toohey et al., 2020; Bell et al., 2021; Hooshmand Moghadam et al., 2021; Isanejad et al., 2023), 3 studies targeted colorectal cancer survivors (Devin et al., 2016; Devin et al., 2018; Moraitis et al., 2023), and 2 studies included mixed types of cancer survivors (Toohey et al., 2016; Toohey et al., 2018). The exercise duration varied from 3 to 12 weeks, with a frequency of 2–5 days per week, predominantly 3 days per week. The duration of each session ranged from 10 to 75 min (Supplementary Table S2).

### 3.3 Risk of bias assessment

According to the assessment based on Cochrane RoB2, we found that 4 studies (Schmitt et al., 2016; Toohey et al., 2020; Bell et al., 2021; Isanejad et al., 2023) showed some concerns of bias, while 8 studies (Devin et al., 2016; Dolan et al., 2016; Toohey et al., 2016; Devin et al., 2018; Toohey et al., 2018; Northey et al., 2019; Hooshmand Moghadam et al., 2021; Moraitis et al., 2023) were at high risk of bias. In terms of “Bias arising from the randomization process”, 8 studies (Devin et al., 2016; Dolan et al., 2016; Toohey et al., 2016; Devin et al., 2018; Toohey et al., 2018; Northey et al., 2019; Hooshmand Moghadam et al., 2021; Moraitis et al., 2023) presented some concerns due to the lack of clear descriptions of the randomization process and allocation concealment methods. For “Bias due to deviations from intended intervention”, all studies (Devin et al., 2016; Dolan et al., 2016; Schmitt et al., 2016; Toohey et al., 2016; Devin et al., 2018; Toohey et al., 2018; Northey et al., 2019; Toohey et al., 2020; Bell et al., 2021; Hooshmand Moghadam et al., 2021; Isanejad et al., 2023; Moraitis et al., 2023) might have been subjected to performance bias as neither participants nor researchers implemented blinding. Regarding “Bias due to missing outcome data”, 1 study (Moraitis et al., 2023) exhibited some concerns as the proportion of missing participants exceeded 10%. In “Bias in measurement of the outcome”, 8 studies (Devin et al., 2016; Dolan et al., 2016; Schmitt et al., 2016; Toohey et al., 2016; Devin et al., 2018; Toohey et al., 2018; Northey et al., 2019; Bell et al., 2021) had some concerns mainly because it was unclear whether the outcome assessors were blinded to group allocation. Concerning “Bias in selection of the reported result”, 3 studies (Dolan et al., 2016; Devin et al., 2018; Isanejad et al., 2023) showed some concerns due to the absence of information on whether the study protocol was pre-registered (Supplementary Table S3).

### 3.4 Meta-analysis

#### 3.4.1 Cardiopulmonary function

##### 3.4.1.1 VO_2_ peak

**FIGURE 2:**
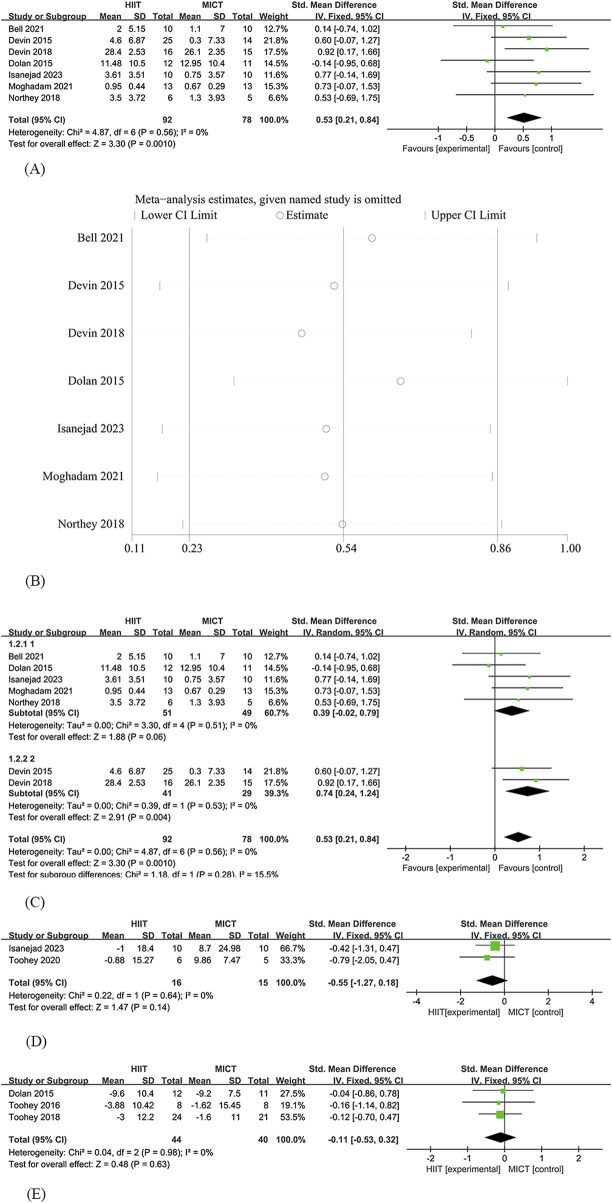
HIIT versus MCIT with Cardiopulmonary function **(A)** The forest plot of the VO_2_ peak **(B)** Sensitivity analysis of the VO_2_ peak **(C)** Subgroup analysis of the VO_2_peak **(D)** The forest plot of the Heart Rate **(E)** The forest plot of the Resting Heart Rate.

Since among the 12 studies, only 7 involved the analysis of VO_2_ Peak, Therefore, Seven studies (Devin et al., 2016; Dolan et al., 2016; Devin et al., 2018; Northey et al., 2019; Bell et al., 2021; Hooshmand Moghadam et al., 2021; Isanejad et al., 2023) were included in the analysis of the effects of HIIT and MICT on VO_2_ Peak in cancer survivors. Among these, 92 participants were assigned to the HIIT group and 78 participants to the MICT group. The analysis revealed a significant difference in VO_2_ Peak between HIIT and MICT [SMD = 0.53, 95% CI (0.21, 0.84), Z = 3.30, P = 0.001], with no heterogeneity observed (I^2^ = 0%). The effect of the HIIT group was significantly greater than that of the MICT group (Figure 2A).

##### 3.4.1.2 Sensitivity analysis

Allows for the evaluation of the robustness and reliability of results by systematically removing individual studies to assess their impact on the overall findings. In the sensitivity analysis, we found that even after excluding individual studies, the effect of HIIT and MICT on VO_2_ Peak in cancer survivors remained significant, and the results of the original meta-analysis did not significantly change due to variations in the number of studies. This indicates the robustness of the results (Figure 2B).

##### 3.4.1.3 Publication bias analysis

To assess publication bias, we performed Egger’s linear regression test. The results showed a P-value of 0.75, which is greater than 0.5, indicating that there is no significant publication bias according to Egger’s linear regression analysis.

##### 3.4.1.4 Subgroup analysis

Five studies (Dolan et al., 2016; Northey et al., 2019; Bell et al., 2021; Hooshmand Moghadam et al., 2021; Isanejad et al., 2023) included breast cancer survivors, totaling 100 subjects. Results showed an improvement in VO_2_ peak in the HIIT group [SMD = 0.39, 95% CI (−0.02, 0.79), Z = 1.88, P = 0.06], the difference did not reach significance and was lower than the overall effect (SMD = 0.53). 2 studies (Devin et al., 2016; Devin et al., 2018) included colorectal cancer survivors, a total of 70 subjects, and showed [SMD = 0.74, 95% CI (0.24, 0.84), Z = 2.91, P = 0.004] (Figure 2C).

##### 3.4.1.5 Heart rate

Two studies (Toohey et al., 2020; Isanejad et al., 2023) were included in the analysis of the effects of HIIT and MICT on Heart Rate in cancer survivors. Among these, 16 participants were assigned to the HIIT group and 15 participants to the MICT group. The analysis found no significant difference in Heart Rate between HIIT and MICT [SMD = −0.55, 95% CI (−1.27, 0.18), Z = 1.47, P = 0.14], with no heterogeneity observed (I^2^ = 0%) (Figure 2D).

##### 3.4.1.6 Resting Heart Rate

Three studies (Dolan et al., 2016; Toohey et al., 2016; Toohey et al., 2018) were included in the analysis of the effects of HIIT and MICT on Resting Heart Rate in cancer survivors. Among these, 44 participants were assigned to the HIIT group and 40 participants to the MICT group. The analysis found no significant difference in Resting Heart Rate between HIIT and MICT [SMD = −0.11, 95% CI (−0.53, 0.32), Z = 0.48, P = 0.63], with no heterogeneity observed (I^2^ = 0%) (Figure 2E).

#### 3.4.2 Body composition

##### 3.4.2.1 Body mass

**FIGURE 3:**
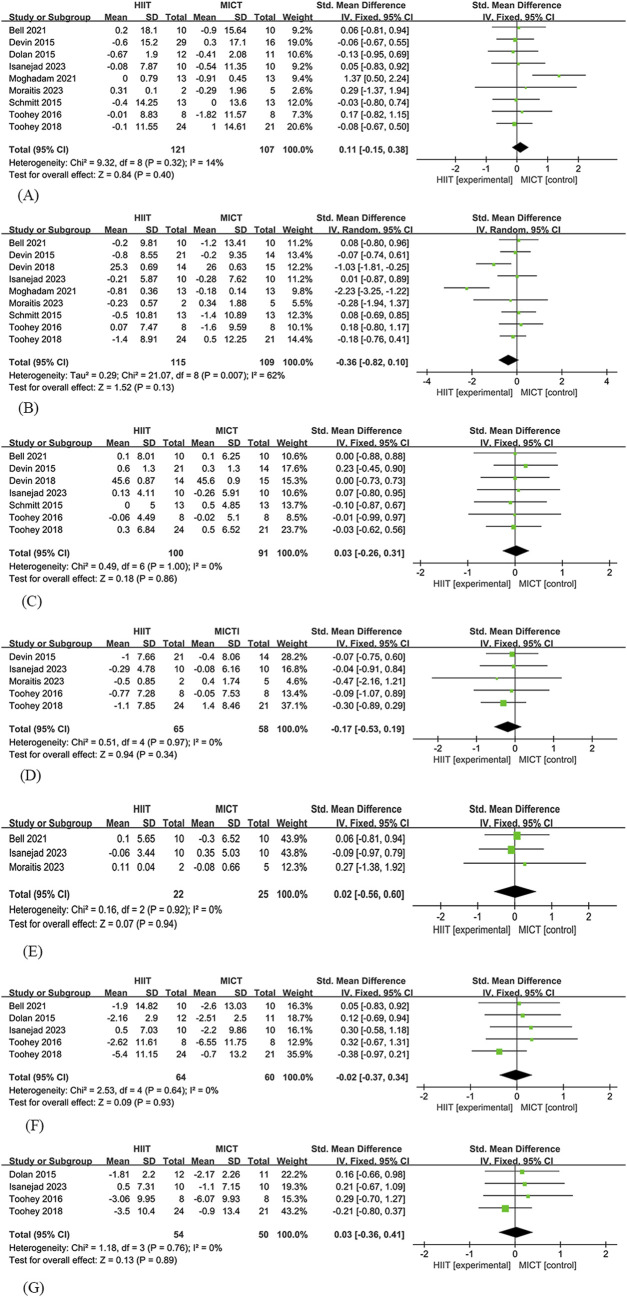
HIIT versus MCIT with Body composition **(A)** The forest plot of the Body mass **(B)** The forest plot of the Total fat mass **(C)** The forest plot of the Lean body mass **(D)** The forest plot of the Fat percentage **(E)** The forest plot of the Body Mass Index **(F)** The forest plot of the Waist circumference **(G)** The forest plot of the Hip circumference.

Nine studies (Devin et al., 2016; Dolan et al., 2016; Schmitt et al., 2016; Toohey et al., 2016; Toohey et al., 2018; Bell et al., 2021; Hooshmand Moghadam et al., 2021; Isanejad et al., 2023; Moraitis et al., 2023) were included in the analysis of the effects of HIIT and MICT on body mass in cancer survivors. Among these, 121 participants were assigned to the HIIT group and 107 participants to the MICT group. The analysis found no significant difference in body mass between HIIT and MICT [SMD = 0.11, 95% CI (−0.15, 0.38), Z = 0.84, P = 0.4], with low heterogeneity observed (I^2^ = 14%) (Figure 3A).

##### 3.4.2.2 Total fat mass

Nine studies (Devin et al., 2016; Schmitt et al., 2016; Toohey et al., 2016; Devin et al., 2018; Toohey et al., 2018; Bell et al., 2021; Hooshmand Moghadam et al., 2021; Isanejad et al., 2023; Moraitis et al., 2023) were included in the analysis of the effects of HIIT and MICT on total fat mass in cancer survivors. Among these, 115 participants were assigned to the HIIT group and 109 participants to the MICT group. The analysis found no significant difference in total fat mass between HIIT and MICT [SMD = −0.36, 95% CI (−0.82, 0.1), Z = 1.52, P = 0.13], with high heterogeneity observed (I^2^ = 62%) (Figure 3B).

##### 3.4.2.3 Lean body mass

Seven studies (Devin et al., 2016; Schmitt et al., 2016; Toohey et al., 2016; Devin et al., 2018; Toohey et al., 2018; Bell et al., 2021; Isanejad et al., 2023) were included in the analysis of the effects of HIIT and MICT on lean body mass in cancer survivors. Among these, 100 participants were assigned to the HIIT group and 91 participants to the MICT group. The analysis found no significant difference in lean body mass between HIIT and MICT [SMD = −0.03, 95% CI (−0.26, 0.31), Z = 0.18, P = 0.86], with no heterogeneity observed (I^2^ = 0%) (Figure 3C).

##### 3.4.2.4 Fat percentage

Five studies (Devin et al., 2016; Toohey et al., 2016; Toohey et al., 2018; Isanejad et al., 2023; Moraitis et al., 2023) were included in the analysis of the effects of HIIT and MICT on fat percentage in cancer survivors. Among these, 65 participants were assigned to the HIIT group and 58 participants to the MICT group. The analysis found no significant difference in fat percentage between HIIT and MICT [SMD = −0.17, 95% CI (−0.53, 0.19), Z = 0.94, P = 0.34], with no heterogeneity observed (I^2^ = 0%) (Figure 3D).

##### 3.4.2.5 Body mass index

Three studies (Bell et al., 2021; Isanejad et al., 2023; Moraitis et al., 2023) were included in the analysis of the effects of HIIT and MICT on BMI(Body Mass Index) in cancer survivors. Among these, 22 participants were assigned to the HIIT group and 25 participants to the MICT group. The analysis found no significant difference in BMI between HIIT and MICT [SMD = 0.02, 95% CI (−0.56, 0.6), Z = 0.07, P = 0.94], with no heterogeneity observed (I^2^ = 0%) (Figure 3E).

##### 3.4.2.6 Waist circumference

Five studies (Dolan et al., 2016; Toohey et al., 2016; Toohey et al., 2018; Bell et al., 2021; Isanejad et al., 2023) were included in the analysis of the effects of HIIT and MICT on waist circumference in cancer survivors. Among these, 64 participants were assigned to the HIIT group and 60 participants to the MICT group. The analysis found no significant difference in waist circumference between HIIT and MICT [SMD = −0.02, 95% CI (−0.37, 0.34), Z = 0.09, P = 0.93], with no heterogeneity observed (I^2^ = 0%) (Figure 3F).

##### 3.4.2.7 Hip circumference

Four studies (Dolan et al., 2016; Toohey et al., 2016; Toohey et al., 2020; Isanejad et al., 2023) were included in the analysis of the effects of HIIT and MICT on hip circumference in cancer survivors. Among these, 54 participants were assigned to the HIIT group and 50 participants to the MICT group. The analysis found no significant difference in hip circumference between HIIT and MICT [SMD = 0.03, 95% CI (−0.36, 0.41), Z = 0.13, P = 0.89], with no heterogeneity observed (I^2^ = 0%) (Figure 3G).

#### 3.4.3 Physical function

##### 3.4.3.1 STS

**FIGURE 4:**
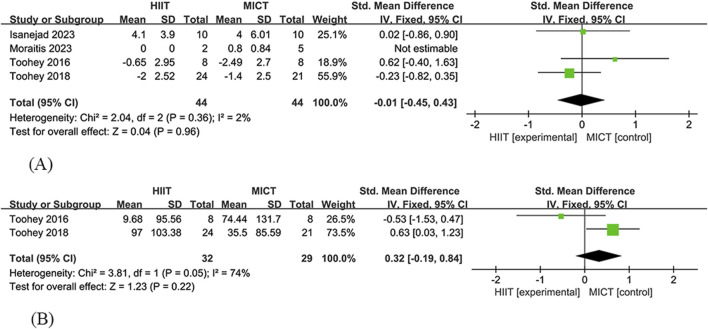
HIIT versus MCIT with Physical function **(A)** The forest plot of the STS **(B)** The forest plot of the 6MWT.

Four studies (Toohey et al., 2016; Toohey et al., 2018; Isanejad et al., 2023; Moraitis et al., 2023) were included in the analysis of the effect of HIIT and MICT on the Sit-to-Stand (STS) test in cancer survivors. The HIIT group consisted of 44 participants, while the MICT group had 44 participants. The study found no significant difference in STS between HIIT and MICT [SMD = −0.01, 95% CI (−0.45, 0.43), Z = 0.04, P = 0.96], showing low heterogeneity (I^2^ = 2%) (Figure 4A).

##### 3.4.3.2 6MWT

Two studies (Toohey et al., 2016; Toohey et al., 2018) were included in the analysis of the effect of HIIT and MICT on the 6-Minute Walk Test (6MWT) in cancer survivors. The HIIT group consisted of 32 participants, while the MICT group had 29 participants. The study found no significant difference in 6MWT between HIIT and MICT [SMD = 0.32, 95% CI (−0.19, 0.84), Z = 1.23, P = 0.22], showing high heterogeneity (I^2^ = 74%) (Figure 4B).

## 4 Discussion

The primary objective of this systematic review and meta-analysis was to analyze the effect of HIIT and MICT on VO_2_ peak in cancer survivors and evaluate which exercise modality is more effective. The secondary objective was to analyze the impact of HIIT and MICT on body composition and physical function in cancer survivors. The results of this study showed that, compared to MICT, HIIT led to a statistically significant increase in VO_2_ peak in cancer survivors. However, no statistically significant differences were found between HIIT and MICT in terms of body composition and physical function. It is important to note that the studies included in this meta-analysis focused on breast and colorectal cancer survivors, with only 2 studies including mixed cancer types. Although HIIT has shown significant benefits in improving VO_2_ peak in breast and colorectal cancer patients, there may be differences in adaptations to high-intensity training in other cancer types due to physical status limitations.

Cardiopulmonary health, with VO_2_ peak as the core indicator, is a key physiological measure in the rehabilitation process of cancer survivors. It is closely associated with their quality of life, treatment tolerance, recurrence risk, and long-term survival rates. Lower levels of VO_2_ peak are linked to an increased cancer-specific mortality rate, making the enhancement of cardiopulmonary function a critical factor in reducing cancer-specific mortality. This emphasizes the importance of cardiopulmonary health in cancer survivors (Zhang et al., 2014; Lakoski et al., 2015; Schmid and Leitzmann, 2015).

Although this meta-analysis did not directly assess the effect of HIIT on cancer recurrence or survival, existing studies suggest that improvements in VO_2_ peak may be strongly associated with mortality. For example, a cohort study by Lakoski et al. demonstrated a strong inverse association between cardiorespiratory fitness and colorectal cancer with a corrected hazard ratio (HR) of 0.56 (95% CI: 0.36–0.87) for colorectal cancer in men with high cardiorespiratory fitness compared with low cardiorespiratory fitness. High cardiorespiratory fitness reduced the risk of colorectal cancer by 44% compared to low cardiorespiratory fitness (Lakoski et al., 2015). In addition, a study by Kenfield et al. found that physical activity was associated with lower cancer mortality among men with cancer. Men who performed ≥3 h of vigorous activity per week had a 49% lower risk of all-cause mortality (HR, 0.51; 95% CI, 0.36–0.72) compared with shorter durations at an easy walking pace (Kenfield et al., 2011). Similarly, Zhang et al. found that maintaining or improving cardiorespiratory fitness may reduce the risk of cancer death. Loss of cardiorespiratory fitness was associated with an increased risk of premature death from cancer in men. In terms of public health messages, health and medical professionals should advise individuals to engage in regular physical activity to gain or maintain cardiorespiratory fitness (Zhang et al., 2014). Although these studies did not directly compare HIIT with MICT, their results support that high-intensity exercise may improve long-term prognosis through more efficient physiological adaptive mechanisms.

This study primarily found that HIIT improved cardiopulmonary health in cancer survivors compared to the MICT group. This finding is consistent with some research results. Several studies have shown that cancer survivors benefit more from HIIT than from MICT in terms of VO_2_ peak improvement (Rognmo et al., 2004; Wisløff et al., 2007). For example, Isanejad’s study found that MICT seemed to have no significant effect on VO_2_ peak, whereas HIIT was superior in improving VO_2_ peak (mean difference = 2.974 mL/kg/min, 95% CI: -0.188 to 6.135, p = 0.005) (Isanejad et al., 2023). Devin’s research showed that the absolute (p = 0.016) and relative (p = 0.021) mean changes in VO_2_ peak in the HIIT group were significantly greater than in the MICT group (Devin et al., 2016). Billat et al. found that intermittent running with high-intensity components provided greater training stimuli compared to continuous running and might lead to more significant improvements in VO_2_ peak associated with oxygen consumption post-exercise (Billat et al., 2000).

However, there are also inconsistent findings. Mugele et al. concluded that when comparing HIIT to conventional treatments, the intervention group showed an increase in VO_2_ peak, but no significant difference was found between HIIT and MICT (Mugele et al., 2019). The BELL study found that HIIT did not lead to a greater VO_2_ peak improvement than MICT; however, they encouraged future studies with higher intensity and frequency of training (Bell et al., 2021). The discrepancies between studies could be attributed to heterogeneity in the study populations (different cancer types, treatment stages, and comorbidities), variations in intervention protocols (different HIIT intensities, durations of intervals, and low-intensity or insufficient time in MICT), differences in adherence, and variations in measurement methods.

Based on the literature, it is hypothesized that the improvement of VO_2_ peak by HIIT may be related to the following mechanisms: (1) HIIT activates the AMPK-PGC-1α pathway and promotes the density and function of skeletal muscle mitochondria. Studies have shown that HIIT significantly increases AMPK phosphorylation and PGC-1α mRNA expression in skeletal muscle, promoting mitochondrial biosynthesis. For example, Broome et al. reported enhanced PGC-1α expression after HIIT, which correlated with increased peak power output, an indirect marker of increased peak VO_2_ (Broome et al., 2022). This is supported by animal studies, which showed that the HIIT group was higher than the MICT group (P = 0.008), suggesting that HIIT significantly increased the expression of AMPK and PGC-1α more than MICT (Pirani et al., 2023). (2) Catecholamine-mediated cardiorespiratory adaptations. HIIT significantly increases plasma catecholamine (epinephrine and norepinephrine) levels during and after exercise, and these catecholamines have been associated with improved cardiovascular responses through β-adrenergic signaling. For example, Williams et al. demonstrated that exercise-induced catecholamine release enhances myocardial β-adrenergic receptor density, which may improve oxygen delivery and utilization during peak exercise (Williams et al., 1985). It should be clarified that the above mechanisms have not been directly verified in the present study.

We observed no statistically significant differences between HIIT and MICT in body composition (Body mass, Total fat mass, Lean body mass, Fat percentage, BMI, Waist circumference, and Hip circumference). Previous studies have indicated that exercise alone may not lead to weight loss, but preventing weight gain could be essential (Jakicic and Otto, 2005; Franz et al., 2007). In a randomized controlled trial, Isanejad et al. found no significant differences between the two groups in fat mass (p = 0.255), waist circumference (p = 0.397), hip circumference (p = 0.528), body weight (p = 0.613), and BMI (p = 0.497) after intervention (Isanejad et al., 2023). For measurements of lean body mass, fat mass, or fat percentage, no significant differences were observed between HIIT and MICT. However, while the HIIT group showed a greater average reduction in fat mass compared to the MICT group, the difference was not statistically significant (p = 0.060) (Devin et al., 2016).

We observed no significant differences between HIIT and MICT in physical function (STS, 6MWT). Consistent with our findings, Isanejad used the STS test to assess physical function and found no significant difference between the HIIT and MICT groups (p = 0.266) (Isanejad et al., 2023). However, Toohey’s study found that participants in the HIIT group achieved a greater effect in the STS test compared to the MICT group, suggesting improvements in lower limb strength (Toohey et al., 2016; Toohey et al., 2018). In the STS test, improvements were observed in both the HIIT group (20%) and the MICT group (9.5%), highlighting an increase in calf strength among those who completed the HIIT program. However, due to the small sample size, this difference was not statistically significant (Toohey et al., 2016). The lack of significant differences between HIIT and MICT in body composition and physical function in cancer survivors may stem from multiple factors such as energy expenditure balance, metabolic disturbances, insufficient intervention duration, and cancer-specific pathological mechanisms (Boutcher, 2011; Pedersen and Saltin, 2015; Graßmann et al., 2017; Maestroni et al., 2020).

This study systematically integrates RCTs and is the first comprehensive assessment of the effects of HIIT and MICT on cancer survivors’ cardiopulmonary function (with VO_2_ peak as the core indicator), body composition, and physical function. The limitations of the study include: (1) the limited number of included studies and participants, which may lead to unreliable study results; (2) contains study heterogeneity, including inconsistencies in HIIT and MICT intervention protocols (including intensity, frequency vs. duration), cancer type, and other factors, as well as errors inherent in different measurement tools. The uneven distribution of cancer types in the study population, which was dominated by breast and colorectal cancers, may limit the applicability of the findings to other cancer subgroups; (3) potential publication bias and the lack of long-term follow-up data; (4) the inability to implement participant blinding due to the nature of the study type, making performance bias unavoidable in all trials; (5) the lack of subgroup analysis due to the limited number of studies, preventing understanding of the differences in effects across different populations, interventions, and durations; and (6) another limitation of this study is that survival or recurrence rates were not directly analyzed. Although VO_2_ peak is an independent predictor of cancer prognosis, its clinical translation still needs to be validated by randomized controlled trials (RCTs) with long-term follow-up.

Future research should aim to expand sample sizes, include subgroups of different cancer types, stages, and treatment phases, standardize interventions, and further optimize the precision and universality of exercise rehabilitation strategies for different cancer subtypes. Long-term follow-up should be established to track the effects of HIIT on cancer survivors’ cardiopulmonary function and recurrence rates. Modern techniques such as remote monitoring and AI personalized prescriptions should be utilized to enhance the feasibility and clinical translational value of interventions.

## 5 Conclusion

This study found that for cancer survivors, HIIT appeared superior to MICT in enhancing VO_2_ peak and, consequently, cardiopulmonary function in breast and colorectal cancer survivors. However, further studies are needed to validate its safety and efficacy in other cancer populations, particularly those with higher vulnerability to exercise-induced adverse events. There were no significant differences between the two training modes in terms of body composition and physical function indicators, which may be related to factors such as energy metabolism balance and insufficient intervention duration. It is important to note that the conclusions are based on a limited number of studies, and more research is needed in the future to explore the effects of HIIT and MICT on cancer survivors. This will help in more accurately exploring personalized exercise prescriptions and optimizing cancer rehabilitation outcomes.

## Data Availability

The original contributions presented in the study are included in the article/Supplementary Material, further inquiries can be directed to the corresponding author.

## Abbreviations

RCTs, randomized controlled trials; HIIT, high-intensity interval training; MICT, moderate-intensity continuous training; VO_2_ peak, peak oxygen uptake; SMD, standardized mean difference; BMI, body mass index; STS, sit-to-stand test; 6MWT, 6-minute walk test.

## References

[B1] Bartlett J. D. Close G. L. MacLaren D. P. Gregson W. Drust B. Morton J. P. (2011). High-intensity interval running is perceived to be more enjoyable than moderate-intensity continuous exercise: implications for exercise adherence. J. Sports Sci. 29 (6), 547–553. 10.1080/02640414.2010.545427 21360405

[B2] Batacan R. B. Jr. Duncan M. J. Dalbo V. J. Tucker P. S. Fenning A. S. (2017). Effects of high-intensity interval training on cardiometabolic health: a systematic review and meta-analysis of intervention studies. Br. J. Sports Med. 51 (6), 494–503. 10.1136/bjsports-2015-095841 27797726

[B3] Bell R. A. Baldi J. C. Jones L. M. (2021). Additional cardiovascular fitness when progressing from moderate-to high-intensity exercise training in previously trained breast cancer survivors. Support. Care Cancer 29 (11), 6645–6650. 10.1007/s00520-021-06259-w 33956212

[B4] Billat V. L. Slawinski J. Bocquet V. Demarle A. Lafitte L. Chassaing P. (2000). Intermittent runs at the velocity associated with maximal oxygen uptake enables subjects to remain at maximal oxygen uptake for a longer time than intense but submaximal runs. Eur. J. Appl. Physiol. 81 (3), 188–196. 10.1007/s004210050029 10638376

[B5] Boutcher S. H. (2011). High-intensity intermittent exercise and fat loss. J. Obes. 2011, 868305. 10.1155/2011/868305 21113312 PMC2991639

[B6] Bray F. Laversanne M. Sung H. Ferlay J. Siegel R. L. Soerjomataram I. (2024). Global cancer statistics 2022: GLOBOCAN estimates of incidence and mortality worldwide for 36 cancers in 185 countries. CA A Cancer J. Clin. 74 (3), 229–263. 10.3322/caac.21834 38572751

[B7] Broome S. C. Pham T. Braakhuis A. J. Narang R. Wang H. W. Hickey A. J. R. (2022). MitoQ supplementation augments acute exercise-induced increases in muscle PGC1α mRNA and improves training-induced increases in peak power independent of mitochondrial content and function in untrained middle-aged men. Redox Biol. 53, 102341. 10.1016/j.redox.2022.102341 35623315 PMC9142706

[B8] Cariolou M. Abar L. Aune D. Balducci K. Becerra-Tomás N. Greenwood D. C. (2023). Postdiagnosis recreational physical activity and breast cancer prognosis: global Cancer Update Programme (CUP Global) systematic literature review and meta-analysis. Int. J. Cancer 152 (4), 600–615. 10.1002/ijc.34324 36279903 PMC10091720

[B9] Chen K. Guan H. Sun M. Zhang Y. Zhong W. Guo X. (2024). Effects of physical activity on cardiotoxicity and cardio respiratory function in cancer survivors undergoing chemotherapy: a systematic review and meta-analysis. Integr. Cancer Ther. 23, 1176. 10.1177/15347354241291176 PMC11487611 39415360

[B11] Costa I. Bittar C. S. Fonseca S. M. R. Carolina M. P. D E. S. Dos Santos Rehder M. H. H. Rizk S. I. (2020). Brazilian cardio-oncology: the 10-year experience of the Instituto do Cancer do Estado de Sao Paulo. BMC Cardiovasc. Disord. 20 (1), 206. 10.1186/s12872-020-01471-8 32345217 PMC7189468

[B12] Cramer H. Lauche R. Klose P. Lange S. Langhorst J. Dobos G. J. (2017). Yoga for improving health-related quality of life, mental health and cancer-related symptoms in women diagnosed with breast cancer. Cochrane Database Syst. Rev. 1 (1), Cd010802. 10.1002/14651858.CD010802.pub2 28045199 PMC6465041

[B13] Daum C. W. Cochrane S. K. Fitzgerald J. D. Johnson L. Buford T. W. (2016). Exercise interventions for preserving physical function among cancer survivors in middle to late life. J. Frailty Aging 5 (4), 214–224. 10.14283/jfa.2016.92 27883168

[B14] Denlinger C. S. Barsevick A. M. (2009). The challenges of colorectal cancer survivorship. J. Natl. Compr. Canc Netw. 7 (8), 883–893. 10.6004/jnccn.2009.0058 19755048 PMC3110673

[B15] Denlinger C. S. Carlson R. W. Are M. Baker K. S. Davis E. Edge S. B. (2014). Survivorship: introduction and definition. Clinical practice guidelines in oncology. J. Natl. Compr. Canc. Netw. 12 (1), 34–45. 10.6004/jnccn.2014.0005 24453291 PMC4465253

[B16] Devin J. L. Jenkins D. G. Sax A. T. Hughes G. I. Aitken J. F. Chambers S. K. (2018). Cardiorespiratory fitness and body composition responses to different intensities and frequencies of exercise training in colorectal cancer survivors. Clin. Colorectal Cancer 17 (2), e269–e279. 10.1016/j.clcc.2018.01.004 29397328

[B17] Devin J. L. Sax A. T. Hughes G. I. Jenkins D. G. Aitken J. F. Chambers S. K. (2016). The influence of high-intensity compared with moderate-intensity exercise training on cardiorespiratory fitness and body composition in colorectal cancer survivors: a randomised controlled trial. J. Cancer Surviv. 10 (3), 467–479. 10.1007/s11764-015-0490-7 26482384

[B18] Dolan L. B. Campbell K. Gelmon K. Neil-Sztramko S. Holmes D. McKenzie D. C. (2016). Interval versus continuous aerobic exercise training in breast cancer survivors--a pilot RCT. Support Care Cancer 24 (1), 119–127. 10.1007/s00520-015-2749-y 25957010

[B19] Elshahat S. Treanor C. Donnelly M. (2021). Factors influencing physical activity participation among people living with or beyond cancer: a systematic scoping review. Int. J. Behav. Nutr. Phys. Act. 18 (1), 50. 10.1186/s12966-021-01116-9 33823832 PMC8025326

[B20] Foulkes S. J. Howden E. J. Haykowsky M. J. Antill Y. Salim A. Nightingale S. S. (2023). Exercise for the prevention of anthracycline-induced functional disability and cardiac dysfunction: the BREXIT study. Brexit Study. 147 (7)**,** 532–545. 10.1161/CIRCULATIONAHA.122.062814 36342348

[B21] Franz M. J. VanWormer J. J. Crain A. L. Boucher J. L. Histon T. Caplan W. (2007). Weight-loss outcomes: a systematic review and meta-analysis of weight-loss clinical trials with a minimum 1-year follow-up. J. Am. Diet. Assoc. 107 (10), 1755–1767. 10.1016/j.jada.2007.07.017 17904936

[B22] Furmaniak A. C. Menig M. Markes M. H. (2016). Exercise for women receiving adjuvant therapy for breast cancer. Cochrane Database Syst. Rev. 9 (9), Cd005001. 10.1002/14651858.CD005001.pub3 27650122 PMC6457768

[B23] Gibala M. J. Gillen J. B. Percival M. E. (2014). Physiological and health-related adaptations to low-volume interval training: influences of nutrition and sex. Sports Med. 44 (Suppl. 2), S127–S137. 10.1007/s40279-014-0259-6 25355187 PMC4213388

[B24] Gillen J. B. Gibala M. J. (2014). Is high-intensity interval training a time-efficient exercise strategy to improve health and fitness? Appl. Physiol. Nutr. Metab. 39 (3), 409–412. 10.1139/apnm-2013-0187 24552392

[B25] Graßmann S. Wirsching J. Eichelmann F. Aleksandrova K. (2017). Association between peripheral adipokines and inflammation markers: a systematic review and meta-analysis. Obes. (Silver Spring) 25 (10), 1776–1785. 10.1002/oby.21945 28834421

[B26] Guo Z. Saw P. E. Jon S. (2024). Non-invasive physical stimulation to modulate the tumor microenvironment: unveiling a New frontier in cancer therapy. Bio. Integr. 5 (1), 1–14. 10.15212/bioi-2024-0012

[B27] He S. Xia C. Li H. Cao M. Yang F. Yan X. (2024). Cancer profiles in China and comparisons with the USA: a comprehensive analysis in the incidence, mortality, survival, staging, and attribution to risk factors. Sci. China Life Sci. 67 (1), 122–131. 10.1007/s11427-023-2423-1 37755589

[B28] Hilfiker R. Meichtry A. Eicher M. Nilsson Balfe L. Knols R. H. Verra M. L. (2018). Exercise and other non-pharmaceutical interventions for cancer-related fatigue in patients during or after cancer treatment: a systematic review incorporating an indirect-comparisons meta-analysis. Br. J. Sports Med. 52 (10), 651–658. 10.1136/bjsports-2016-096422 28501804 PMC5931245

[B29] Hooshmand Moghadam B. Golestani F. Bagheri R. Cheraghloo N. Eskandari M. Wong A. (2021). The effects of high-intensity interval training vs. Moderate-intensity continuous training on inflammatory markers, body composition, and physical fitness in overweight/obese survivors of breast cancer: a randomized controlled clinical trial. Cancers (Basel) 13 (17), 4386. 10.3390/cancers13174386 34503198 PMC8430701

[B30] Isanejad A. Nazari S. Gharib B. Motlagh A. G. (2023). Comparison of the effects of high-intensity interval and moderate-intensity continuous training on inflammatory markers, cardiorespiratory fitness, and quality of life in breast cancer patients. J. Sport Health Sci. 12 (6), 674–689. 10.1016/j.jshs.2023.07.001 37423313 PMC10658315

[B31] Jakicic J. M. Otto A. D. (2005). Physical activity considerations for the treatment and prevention of obesity. Am. J. Clin. Nutr. 82 (1 Suppl. l), 226S–229s. 10.1093/ajcn/82.1.226S 16002826

[B32] Jereczek-Fossa B. A. Marsiglia H. R. Orecchia R. (2002). Radiotherapy-related fatigue. Crit. Rev. Oncol. Hematol. 41 (3), 317–325. 10.1016/s1040-8428(01)00143-3 11880207

[B33] Kenfield S. A. Stampfer M. J. Giovannucci E. Chan J. M. (2011). Physical activity and survival after prostate cancer diagnosis in the health professionals follow-up study. J. Clin. Oncol. 29 (6), 726–732. 10.1200/jco.2010.31.5226 21205749 PMC3056656

[B34] Lakoski S. G. Willis B. L. Barlow C. E. Leonard D. Gao A. Radford N. B. (2015). Midlife cardiorespiratory fitness, incident cancer, and survival after cancer in men: the cooper center longitudinal study. JAMA Oncol. 1 (2), 231–237. 10.1001/jamaoncol.2015.0226 26181028 PMC5635343

[B35] Lee K. Kang I. Mack W. J. Mortimer J. Sattler F. Salem G. (2019). Feasibility of high intensity interval training in patients with breast Cancer undergoing anthracycline chemotherapy: a randomized pilot trial. BMC Cancer 19 (1), 653. 10.1186/s12885-019-5887-7 31269914 PMC6610838

[B36] Luo Z. Mei J. Wang X. Wang R. He Z. Geffen Y. (2024). Voluntary exercise sensitizes cancer immunotherapy via the collagen inhibition-orchestrated inflammatory tumor immune microenvironment. Cell Rep. 43 (9), 114697. 10.1016/j.celrep.2024.114697 39217611

[B37] Maestroni L. Read P. Bishop C. Turner A. (2020). Strength and power training in rehabilitation: underpinning principles and practical strategies to return athletes to high performance. Sports Med. 50 (2), 239–252. 10.1007/s40279-019-01195-6 31559567

[B38] Matthews H. K. Bertoli C. de Bruin R. A. M. (2022). Cell cycle control in cancer. Nat. Rev. Mol. Cell Biol. 23 (1), 74–88. 10.1038/s41580-021-00404-3 34508254

[B39] Milanović Z. Sporiš G. Weston M. (2015). Effectiveness of high-intensity interval training (hit) and continuous endurance training for VO2max improvements: a systematic review and meta-analysis of controlled trials. Sports Med. 45 (10), 1469–1481. 10.1007/s40279-015-0365-0 26243014

[B40] Mitchell T. Barlow C. E. (2011). Review of the role of exercise in improving quality of life in healthy individuals and in those with chronic diseases. Curr. Sports Med. Rep. 10 (4), 211–216. 10.1249/JSR.0b013e318223cc9e 23531896

[B41] Mokashi A. Bhatia N. M. (2024). Integrated network ethnopharmacology, molecular docking, and ADMET analysis strategy for exploring the anti-breast cancer activity of ayurvedic botanicals targeting the progesterone receptor. BIO Integr. 5 (1). 10.15212/bioi-2024-0066

[B42] Moraitis A. M. Rose N. B. Johnson A. F. Dunston E. R. Garrido-Laguna I. Hobson P. (2023). Feasibility and acceptability of an mHealth, home-based exercise intervention in colorectal cancer survivors: a pilot randomized controlled trial. PLoS One 18 (6), e0287152. 10.1371/journal.pone.0287152 37347792 PMC10286977

[B43] Mugele H. Freitag N. Wilhelmi J. Yang Y. Cheng S. Bloch W. (2019). High-intensity interval training in the therapy and aftercare of cancer patients: a systematic review with meta-analysis. J. Cancer Surviv. 13 (2), 205–223. 10.1007/s11764-019-00743-3 30806875

[B44] Mustian K. M. Alfano C. M. Heckler C. Kleckner A. S. Kleckner I. R. Leach C. R. (2017). Comparison of pharmaceutical, psychological, and exercise treatments for cancer-related fatigue: a meta-analysis. JAMA Oncol. 3 (7), 961–968. 10.1001/jamaoncol.2016.6914 28253393 PMC5557289

[B45] Nakano J. Hashizume K. Fukushima T. Ueno K. Matsuura E. Ikio Y. (2018). Effects of aerobic and resistance exercises on physical symptoms in cancer patients: a meta-analysis. Integr. Cancer Ther. 17 (4), 1048–1058. 10.1177/1534735418807555 30352523 PMC6247562

[B46] Northey J. M. Pumpa K. L. Quinlan C. Ikin A. Toohey K. Smee D. J. (2019). Cognition in breast cancer survivors: a pilot study of interval and continuous exercise. J. Sci. Med. Sport 22 (5), 580–585. 10.1016/j.jsams.2018.11.026 30554923

[B47] Pedersen B. K. Saltin B. (2006). Evidence for prescribing exercise as therapy in chronic disease. Scand. J. Med. Sci. Sports 16 (Suppl. 1), 3–63. 10.1111/j.1600-0838.2006.00520.x 16451303

[B48] Pedersen B. K. Saltin B. (2015). Exercise as medicine - evidence for prescribing exercise as therapy in 26 different chronic diseases. Scand. J. Med. Sci. Sports 25 (Suppl. 3), 1–72. 10.1111/sms.12581 26606383

[B49] Pirani H. Bakhtiari A. Amiri B. Salehi O. R. (2023). Beneficial mitochondrial biogenesis in gastrocnemius muscle promoted by high-intensity interval training in elderly female rats. Cell J. 25 (1), 11–16. 10.22074/cellj.2022.557565.1078 36680479 PMC9868433

[B50] Ramos J. S. Dalleck L. C. Tjonna A. E. Beetham K. S. Coombes J. S. (2015). The impact of high-intensity interval training versus moderate-intensity continuous training on vascular function: a systematic review and meta-analysis. Sports Med. 45 (5), 679–692. 10.1007/s40279-015-0321-z 25771785

[B51] Rognmo Ø. Hetland E. Helgerud J. Hoff J. Slørdahl S. A. (2004). High intensity aerobic interval exercise is superior to moderate intensity exercise for increasing aerobic capacity in patients with coronary artery disease. Eur. J. Cardiovasc. Prev. Rehabil. 11 (3), 216–222. 10.1097/01.hjr.0000131677.96762.0c 15179103

[B52] Schmid D. Leitzmann M. F. (2015). Cardiorespiratory fitness as predictor of cancer mortality: a systematic review and meta-analysis. Ann. Oncol. 26 (2), 272–278. 10.1093/annonc/mdu250 25009011

[B53] Schmitt J. Lindner N. Reuss-Borst M. Holmberg H. C. Sperlich B. (2016). A 3-week multimodal intervention involving high-intensity interval training in female cancer survivors: a randomized controlled trial. Physiol. Rep. 4 (3), e12693. 10.14814/phy2.12693 26869680 PMC4758922

[B54] Thum J. S. Parsons G. Whittle T. Astorino T. A. (2017). High-intensity interval training elicits higher enjoyment than moderate intensity continuous exercise. PLoS One 12 (1), e0166299. 10.1371/journal.pone.0166299 28076352 PMC5226715

[B55] Toohey K. Pumpa K. McKune A. Cooke J. DuBose K. D. Yip D. (2018). Does low volume high-intensity interval training elicit superior benefits to continuous low to moderate-intensity training in cancer survivors? World J. Clin. Oncol. 9 (1), 1–12. 10.5306/wjco.v9.i1.1 29468132 PMC5807887

[B56] Toohey K. Pumpa K. McKune A. Cooke J. Welvaert M. Northey J. (2020). The impact of high-intensity interval training exercise on breast cancer survivors: a pilot study to explore fitness, cardiac regulation and biomarkers of the stress systems. BMC Cancer 20 (1), 787. 10.1186/s12885-020-07295-1 32819304 PMC7441660

[B57] Toohey K. Pumpa K. L. Arnolda L. Cooke J. Yip D. Craft P. S. (2016). A pilot study examining the effects of low-volume high-intensity interval training and continuous low to moderate intensity training on quality of life, functional capacity and cardiovascular risk factors in cancer survivors. PeerJ 4, e2613. 10.7717/peerj.2613 27781180 PMC5075690

[B58] von Kemp B. A. Cosyns B. (2023). Radiation-induced pericardial disease: mechanisms, diagnosis, and treatment. Curr. Cardiol. Rep. 25 (10), 1113–1121. 10.1007/s11886-023-01933-3 37584875

[B59] Wallen M. P. Hennessy D. Brown S. Evans L. Rawstorn J. C. Wong Shee A. (2020). High-intensity interval training improves cardiorespiratory fitness in cancer patients and survivors: a meta-analysis. Eur. J. Cancer Care (Engl) 29 (4), e13267. 10.1111/ecc.13267 32469144

[B60] Weston K. S. Wisløff U. Coombes J. S. (2014). High-intensity interval training in patients with lifestyle-induced cardiometabolic disease: a systematic review and meta-analysis. Br. J. Sports Med. 48 (16), 1227–1234. 10.1136/bjsports-2013-092576 24144531

[B61] Williams M. E. Gervino E. V. Rosa R. M. Landsberg L. Young J. B. Silva P. (1985). Catecholamine modulation of rapid potassium shifts during exercise. N. Engl. J. Med. 312 (13), 823–827. 10.1056/nejm198503283121304 2858053

[B62] Wisløff U. Støylen A. Loennechen J. P. Bruvold M. Rognmo Ø. Haram P. M. (2007). Superior cardiovascular effect of aerobic interval training versus moderate continuous training in heart failure patients: a randomized study. Circulation 115 (24), 3086–3094. 10.1161/circulationaha.106.675041 17548726

[B63] Xiong Z. Yuan Y. Yang Y. Qiu B. Bai Y. Wang T. (2024). Optimal exercise dose-response improves health-related quality of life in cancer survivors: a systematic review and Bayesian network meta-analysis of RCTs. Front. Oncol. 14, 1510578. 10.3389/fonc.2024.1510578 39737404 PMC11682983

[B64] Xu H. Guo Z. Li M. Chaves H. V. Pinto V. D.P. T. Filho G. C. (2024). Copper-based nanomaterials for image-guided cancer therapy. BIO Integr. 5 (1), 1–14. 10.15212/bioi-2024-0013

[B65] Xu Y. Zeng Y. Xiao X. Liu H. Zhou B. Luo B. (2023). Targeted imaging of tumor associated macrophages in breast cancer. BIO Integr. 4 (3). 10.15212/bioi-2022-0010

[B66] Yang M. Liu L. Gan C. E. Qiu L. H. Jiang X. J. He X. T. (2020). Effects of home-based exercise on exercise capacity, symptoms, and quality of life in patients with lung cancer: a meta-analysis. Eur. J. Oncol. Nurs. 49, 101836. 10.1016/j.ejon.2020.101836 33120218

[B67] Zhang H. Lv G. Liu S. Liu D. Wu X.-Z. (2022). The artificial intelligence watcher predicts cancer risk by facial features. Traditional Med. Res. 7, 1. 10.53388/TMR20211227255

[B68] Zhang P. Sui X. Hand G. A. Hébert J. R. Blair S. N. (2014). Association of changes in fitness and body composition with cancer mortality in men. Med. Sci. Sports Exerc. 46 (7), 1366–1374. 10.1249/mss.0000000000000225 24276414 PMC4031307

