# Supplementary material for: Effects of high-intensity interval training (HIIT) versus moderate-intensity continuous training (MICT) on cardiopulmonary function, body composition, and physical function in cancer survivors: a meta-analysis of randomized controlled trials
Source: Front Physiol. 2025 Jun 13;16:1594574. doi: 10.3389/fphys.2025.1594574 (PMC12202225; doi:10.3389/fphys.2025.1594574)
Supplement: Supplementary file 1 [file Table1.docx]

Search strategy

**Pubmed（12）**

((((((((((((((((High-Intensity Interval Training) OR (High Intensity Interval Training)) OR (High-Intensity Interval Trainings)) OR (Interval Training, High-Intensity)) OR (Interval Trainings, High-Intensity)) OR (Training, High-Intensity Interval)) OR (Trainings, High-Intensity Interval)) OR (High-Intensity Intermittent Exercise)) OR (Exercise, High-Intensity Intermittent)) OR (Exercises, High-Intensity Intermittent)) OR (High-Intensity Intermittent Exercises)) OR (Sprint Interval Training)) OR (Sprint Interval Trainings)) OR (HIIT))

AND

((((((Moderate-Intensity Continuous Training) OR (moderate-intensity continuous exercise)) OR (Moderate-Intensity Steady-State Training)) OR (continuous aerobic exercise)) OR (moderate endurance training)) OR (MICT)))

AND

(((((((((((((((((Cancer Survivors) OR (Cancer Survivor)) OR (Survivors, Cancer)) OR (Survivors of Childhood Cancer)) OR (Cancer Survivor, Childhood)) OR (Cancer Survivors, Childhood)) OR (Childhood Cancer Survivor)) OR (Childhood Cancer Survivors)) OR (Long-Term Cancer Survivors)) OR (Cancer Survivor, Long-Term)) OR (Cancer Survivors, Long-Term)) OR (Long-Term Cancer Survivor)) OR (Long Term Cancer Survivors)) OR (Survivor, Long-Term Cancer)) OR (Survivors, Long-Term Cancer)) OR (Cancer Survivs Training)) OR TS=(moderate-intensity continuous exercise)) OR TS=(Moderate-Intensity Steady-State Training)) OR TS=(continuous aerobic exercise)) OR TS=(moderate endurance training)) OR TS=(MICT) and Preprint Citation Index (Exclude – Database)

AND

((((((((((((((((TS=(Cancer Survivors)) OR TS=(Cancer Survivor)) OR TS=(Survivors, Cancer)) OR TS=(Survivors of Childhood Cancer)) OR TS=(Cancer Survivor, Childhood)) OR TS=(Cancer Survivors, Childhood)) OR TS=(Childhood Cancer Survivor)) OR TS=(Childhood Cancer Survivors)) OR TS=(Long-Term Cancer Survivors)) OR TS=(Cancer Survivor, Long-Term)) OR TS=(Cancer Survivororship)) OR (Survivorship, Cancer)))

AND

(((((Randomized Controlled Trial) OR (Randomised Controlled Trial)) OR (Randomized Clinical Trial)) OR (Randomised Clinical Trial)) OR (RCT))

**Web of science(19)**

(((((((((((((TS=(High-Intensity Interval Training)) OR TS=(High Intensity Interval Training)) OR TS=(High-Intensity Interval Trainings)) OR TS=(Interval Training, High-Intensity)) OR TS=(Interval Trainings, High-Intensity)) OR TS=(Training, High-Intensity Interval)) OR TS=(Trainings, High-Intensity Interval)) OR TS=(High-Intensity Intermittent Exercise)) OR TS=(Exercise, High-Intensity Intermittent)) OR TS=(Exercises, High-Intensity Intermittent)) OR TS=(High-Intensity Intermittent Exercises)) OR TS=(Sprint Interval Training)) OR TS=(Sprint Interval Trainings)) OR TS=(HIIT) and Preprint Citation Index (Exclude – Database)

AND

(((((TS=(Moderate-Intensity Continuous, Long-Term)) OR TS=(Long-Term Cancer Survivor)) OR TS=(Long Term Cancer Survivors)) OR TS=(Survivor, Long-Term Cancer)) OR TS=(Survivors, Long-Term Cancer)) OR TS=(Cancer Survivorship)) OR TS=(Survivorship, Cancer) and Preprint Citation Index (Exclude – Database)

AND

((((TS=(Randomized Controlled Trial)) OR TS=(Randomised Controlled Trial)) OR TS=(Randomized Clinical Trial)) OR TS=(Randomised Clinical Trial)) OR TS=(RCT) and Preprint Citation Index (Exclude – Database)

**Scopus(13)**

( TITLE-ABS-KEY ( high-intensity AND interval AND training ) OR TITLE-ABS-KEY ( high AND intensity AND interval AND training ) OR TITLE-ABS-KEY ( high-intensity AND interval AND trainings ) OR TITLE-ABS-KEY ( interval AND training, AND high-intensity ) OR TITLE-ABS-KEY ( interval AND trainings, AND high-intensity ) OR TITLE-ABS-KEY ( training, AND high-intensity AND interval ) OR TITLE-ABS-KEY ( trainings, AND high-intensity AND interval ) OR TITLE-ABS-KEY ( high-intensity AND intermittent AND exercise ) OR TITLE-ABS-KEY ( exercise, AND high-intensity AND intermittent ) OR TITLE-ABS-KEY ( exercises, AND high-intensity AND intermittent ) OR TITLE-ABS-KEY ( high-intensity AND intermittent AND exercises ) OR TITLE-ABS-KEY ( sprint AND interval AND training ) OR TITLE-ABS-KEY ( sprint AND interval AND trainings ) OR TITLE-ABS-KEY ( hiit ) )

AND

( TITLE-ABS-KEY ( moderate-intensity AND continuous AND training ) OR TITLE-ABS-KEY ( moderate-intensity AND continuous AND exercise ) OR TITLE-ABS-KEY ( moderate-intensity AND steady-state AND training ) OR TITLE-ABS-KEY ( continuous AND aerobic AND exercise ) OR TITLE-ABS-KEY ( moderate AND endurance AND training ) OR TITLE-ABS-KEY ( mict ) )

AND

( TITLE-ABS-KEY ( cancer AND survivors ) OR TITLE-ABS-KEY ( cancer AND survivor ) OR TITLE-ABS-KEY ( survivors, AND cancer ) OR TITLE-ABS-KEY ( survivors AND of AND childhood AND cancer ) OR TITLE-ABS-KEY ( cancer AND survivor, AND childhood ) OR TITLE-ABS-KEY ( cancer AND survivors, AND childhood ) OR TITLE-ABS-KEY ( childhood AND cancer AND survivor ) OR TITLE-ABS-KEY ( childhood AND cancer AND survivors ) OR TITLE-ABS-KEY ( long-term AND cancer AND survivors ) OR TITLE-ABS-KEY ( cancer AND survivor, AND long-term ) OR TITLE-ABS-KEY ( cancer AND survivors, AND long-term ) OR TITLE-ABS-KEY ( long-term AND cancer AND survivor ) OR TITLE-ABS-KEY ( long AND term AND cancer AND survivors ) OR TITLE-ABS-KEY ( survivor, AND long-term AND cancer ) OR TITLE-ABS-KEY ( survivors, AND long-term AND cancer ) OR TITLE-ABS-KEY ( cancer AND survivorship ) OR TITLE-ABS-KEY ( survivorship, AND cancer ) )

AND

( TITLE-ABS-KEY ( randomized AND controlled AND trial ) OR TITLE-ABS-KEY ( randomised AND controlled AND trial ) OR TITLE-ABS-KEY ( randomized AND clinical AND trial ) OR TITLE-ABS-KEY ( randomised AND clinical AND trial ) OR TITLE-ABS-KEY ( rct ) )

**EMbase(10)**

'high-intensity interval training'/exp OR 'high-intensity interval training' OR ('high intensity' AND interval AND ('training'/exp OR training)) OR 'high intensity interval training':ti,ab,kw OR 'high-intensity interval trainings':ti,ab,kw OR 'interval training, high-intensity':ti,ab,kw OR 'interval trainings, high-intensity':ti,ab,kw OR 'training, high-intensity interval':ti,ab,kw OR 'trainings, high-intensity interval':ti,ab,kw OR 'high-intensity intermittent exercise':ti,ab,kw OR 'exercise, high-intensity intermittent':ti,ab,kw OR 'exercises, high-intensity intermittent':ti,ab,kw OR 'high-intensity intermittent exercises':ti,ab,kw OR 'sprint interval training':ti,ab,kw OR 'sprint interval trainings':ti,ab,kw OR hiit:ti,ab,kw

AND

'moderate-intensity continuous training'/exp OR 'moderate-intensity continuous training' OR ('moderate intensity' AND continuous AND ('training'/exp OR training)) OR 'moderate-intensity continuous exercise':ti,ab,kw OR 'moderate-intensity steady-state training':ti,ab,kw OR 'continuous aerobic exercise':ti,ab,kw OR 'moderate endurance training':ti,ab,kw OR mict:ti,ab,kw

AND

'cancer survivors'/exp OR 'cancer survivors' OR (('cancer'/exp OR cancer) AND ('survivors'/exp OR survivors)) OR 'cancer survivor':ti,ab,kw OR 'survivors, cancer':ti,ab,kw OR 'survivors of childhood cancer':ti,ab,kw OR 'cancer survivor, childhood':ti,ab,kw OR 'cancer survivors, childhood':ti,ab,kw OR 'childhood cancer survivor':ti,ab,kw OR 'childhood cancer survivors':ti,ab,kw OR 'long-term cancer survivors':ti,ab,kw OR 'cancer survivor, long-term':ti,ab,kw OR 'cancer survivors, long-term':ti,ab,kw OR 'long-term cancer survivor':ti,ab,kw OR 'long term cancer survivors':ti,ab,kw OR 'survivor, long-term cancer':ti,ab,kw OR 'survivors, long-term cancer':ti,ab,kw OR 'cancer survivorship':ti,ab,kw OR 'survivorship, cancer':ti,ab,kw

AND

'randomized controlled trial'/exp OR 'randomized controlled trial' OR (randomized AND controlled AND ('trial'/exp OR trial)) OR 'randomised controlled trial':ti,ab,kw OR 'randomized clinical trial':ti,ab,kw OR 'randomised clinical trial':ti,ab,kw OR rct:ti,ab,kw

**Cochrane library(12)**

MeSH descriptor: [High-Intensity Interval Training] explode all trees OR (High Intensity Interval Training):ti,ab,kw OR (High-Intensity Interval Trainings):ti,ab,kw OR (Interval Training, High-Intensity):ti,ab,kw OR (Interval Trainings, High-Intensity):ti,ab,kw OR (Training, High-Intensity Interval):ti,ab,kw OR (Trainings, High-Intensity Interval):ti,ab,kw OR (High-Intensity Intermittent Exercise):ti,ab,kw OR (Exercise, High-Intensity Intermittent):ti,ab,kw OR (Exercises, High-Intensity Intermittent):ti,ab,kw OR (High-Intensity Intermittent Exercises):ti,ab,kw OR (Sprint Interval Training):ti,ab,kw OR (Sprint Interval Trainings):ti,ab,kw OR (HIIT):ti,ab,kw

AND

MeSH descriptor: [Cancer Survivors] explode all trees OR (Cancer Survivor):ti,ab,kw OR (Survivors, Cancer):ti,ab,kw OR (Survivors of Childhood Cancer):ti,ab,kw OR (Cancer Survivor, Childhood):ti,ab,kw OR (Cancer Survivors, Childhood):ti,ab,kw OR (Childhood Cancer Survivor):ti,ab,kw OR (Childhood Cancer Survivors):ti,ab,kw OR (Long-Term Cancer Survivors):ti,ab,kw OR (Cancer Survivor, Long-Term):ti,ab,kw OR (Cancer Survivors, Long-Term):ti,ab,kw OR (Long-Term Cancer Survivor):ti,ab,kw OR (Long Term Cancer Survivors):ti,ab,kw OR (Survivor, Long-Term Cancer):ti,ab,kw OR (Survivors, Long-Term Cancer):ti,ab,kw OR (Cancer Survivorship):ti,ab,kw OR (Survivorship, Cancer):ti,ab,kw

AND

(Randomized Controlled Trial):ti,ab,kw OR (Randomised Controlled Trial):ti,ab,kw OR (Randomized Clinical Trial):ti,ab,kw OR (Randomised Clinical Trial):ti,ab,kw OR (RCT):ti,ab,kw

AND

(Moderate-Intensity Continuous Training):ti,ab,kw OR (moderate-intensity continuous exercise):ti,ab,kw OR (Moderate-Intensity Steady-State Training):ti,ab,kw OR (continuous aerobic exercise):ti,ab,kw OR (moderate endurance training):ti,ab,kw OR (MICT):ti,ab,kw

**EBSCOhost（347）**

TX High-Intensity Interval Training OR TX High Intensity Interval Training OR TX High-Intensity Interval Trainings OR TX Interval Training, High-Intensity OR TX Interval Trainings, High-Intensity OR TX Training, High-Intensity Interval OR TX Trainings, High-Intensity Interval OR TX High-Intensity Intermittent Exercise OR TX Exercise, High-Intensity Intermittent OR TX Exercises, High-Intensity Intermittent OR TX High-Intensity Intermittent Exercises OR TX Sprint Interval Training OR TX Sprint Interval Trainings OR TX HIIT

AND

TX Moderate-Intensity Continuous Training OR TX moderate-intensity continuous exercise OR TX Moderate-Intensity Steady-State Training OR TX continuous aerobic exercise OR TX moderate endurance training OR TX MICT

AND

TX Cancer Survivors OR TX Cancer Survivor OR TX Survivors, Cancer OR TX Survivors of Childhood Cancer OR TX Cancer Survivor, Childhood OR TX Cancer Survivors, Childhood OR TX Childhood Cancer Survivor OR TX Childhood Cancer Survivors OR TX Long-Term Cancer Survivors OR TX Cancer Survivor, Long-Term OR TX Cancer Survivors, Long-Term OR TX Long-Term Cancer Survivor OR TX Long Term Cancer Survivors OR TX Survivor, Long-Term Cancer OR TX Survivors, Long-Term Cancer OR TX Cancer Survivorship OR TX Survivorship, Cancer

AND

TX Randomized Controlled Trial OR TX Randomised Controlled Trial OR TX Randomized Clinical Trial OR TX Randomised Clinical Trial OR TX RCT
