# Supplementary material for: Effects of high-intensity interval training (HIIT) versus moderate-intensity continuous training (MICT) on cardiopulmonary function, body composition, and physical function in cancer survivors: a meta-analysis of randomized controlled trials
Source: Front Physiol. 2025 Jun 13;16:1594574. doi: 10.3389/fphys.2025.1594574 (PMC12202225; doi:10.3389/fphys.2025.1594574)
Supplement: Supplementary file 2 [file Table2.docx]

**Characteristics of the studies**

| Study | Country | Cancer types | Sample size | Sex female:n(%) | Age(mean±sd) | Minutes per session | Days | Duration（weeks） | Intensity | Measurement Instrument |
| --- | --- | --- | --- | --- | --- | --- | --- | --- | --- | --- |
| Bell 2021 | New Zealand | breast cancer survivors | HIIT:10 MICT:10 | HIIT:10(100%) MICT:10(100%) | HIIT:49±4 MICT:51±5 | 33 | 2 | 12 | HIIT:The first 2 weeks of HIIT included four 2-minute high-intensity intervals (70-75% HRR) with 2-minute recovery in between; in the following 10 weeks, the high-intensity intervals were extended to 5 minutes. All sessions included warm-up and cool-down.  MICT:The first four MICT sessions used the same warm-up and cool-down, with continuous stationary cycling at 60% HRR for 14 minutes. In the last 20 sessions, this duration was increased to 25 minutes at 60% HRR. | Peak ­VO2 (mL/kg/min) Peak HR (bpm) HR, heart rate Body mass (kg) Waist circumference(cm) BMI (kg/m2) Total fat mass (kg) Total lean tissue (kg) Appendicular lean muscle index (kg/m2) |
| Devin 2015 | Australia | colorectal cancer survivors | HIE:30 MIE:17 | HIE:12(40%) MIE:9(52.9%) | HIE:61.4±11.1 MIE:61.5±10.8 | HIE:38 MIE:50 | 3 | 4 | HIIT: 85-95% of peak heart rate (HRpeak)  MICT: 70% of peak heart rate (HRpeak) | V̇O2peak (L.min−1) V̇O2peak (ml.kg−1 .min−1) Body mass (kg) Lean mass (kg) Fat mass (kg) Body fat percentage (%) Godin index |
| Devin 2018 | Australia | colorectal cancer survivors | HIIT:18 MICT:19 | HIIT:5(27.8%) MICT:10(52.6%) | HIIT:60.7±11.7 MICT:59.8±11.4 | HIIT:38 MICT:50 | 3 | 8 | HIIT: 10-minute warm-up at 50-70% HRpeak, followed by 4-minute intervals of cycling at 85-95% HRpeak.  MICT: Cycling for 50 minutes at 50-70% HRpeak. | V̇O2peak (ml·kg-1·min-1) Lean mass (kg) Fat mass (kg) |
| Dolan 2015 | Canada | breast cancer survivors | AIT:12 CMT:11 | AIT:12(100%) CMT:11(100%) | AIT:56.2±9 CMT:56.3±9 |  | 3 | 6 | HIIT：70 ~ 100%VO2peak MICT：60 ~ 70%VO2peak | VO2peak Weight Waist Hip HRrest |
| Isanejad 2023 | Iran | breast cancer survivors | HIIT:10 MICT:10 | HIIT:10(100%) MICT:10(100%) | HIIT:44.00±9.14 MICT:46.29±6.29 | HIIT:33 MICT:41 | 3 | 12 | HIIT Warm-up 5 min 50%-60% VO2peak (65%-75% HRpeak) 4 × 4 min 90%VO2peak (95% HRpeak) 3 × 3 min 60%VO2peak (75% HRpeak) Cool-down 3 min 50% 60% VO2peak (65%-75% HRpeak) MICT Warm-up 5 min 50%-60% VO2peak (65% 75% HRpeak) Main training 33 min 60% VO2peak (75% HRpeak) Cool-down 3 min 50%-60% VO2peak (65% 75% HRpeak) | Body weight (kg) BMI (kg/m2) Lean body mass (kg) Fat mass (kg) Fat percent (%) Waist circumference (cm) Hip circumference (cm) Functional capacity (sit-to-stand test) VO2peak (mL/kg/min) HR (beats/min) |
| Moghadam 2021 | Iran | breast cancer survivors | HIIT:13 MICT:13 | HIIT:13(100%) MICT:13(100%) | 57 ± 1.0 | 20-30 | 3 | 12 | MICT : Completed a 5-minute warm-up and cool-down at 50% of participants' peak power, followed by a 20-minute moderate-intensity phase at 55-65% of their peak power. | Fat (g/day) BM, body mass(kg) FM, fat mass(kg) LM, lean mass(kg) VO2peak-Post(mL·kg−1·min−1) |
| Moraitis 2023 | United States | colorectal cancer survivors | HIIT:2 MICT:5 | HIIT:0(0%) MICT:3(43%) | HIIT:39, 44.5 MICT:37 (34, 50) | HIIT:50 MICT:60 | HIIT:4 MICT:5 | 12 | HIIT: Each session includes a 10-minute warm-up with a heart rate of 50-75% of peak HR, followed by five 4-minute intervals at 85-90% of peak HR. After each 4-minute interval, a 4-minute active recovery is performed with heart rate reduced to 50-75% of peak HR.  MICT: Intensity at 50-70% of peak HR. | Weight (kg) BMI (kg/m2) Fat-Free Mass (kg) Fat Mass(kg) Body Fat（%） Fat-Free Mass（%） Handgrip Strength (kg) Sit-to-Stand (seconds) |
| Northey 2018 | Australia | breast cancer survivors | HIIT:6 MICT:5 | HIIT:6(100%) MICT:5(100%) | HIIT:60.3±8.1 MICT:67.8±7 | 20-30 | 3 | 12 | MICT: The cycling group completes a 5-minute warm-up and cool-down at 50% of peak power, followed by a 20-minute steady-state phase at 55-65% of peak power. | VO2Peak, mL kg−1 min−1 |
| Schmitt 2015 | Germany | breast cancer survivors | HIIT:13 LMIE:13 | HIIT:13(100%) LMIE:34(100%) | HIIT:53±8 LMIE:54±9 | HIIT:37 LMIE:75 | 3 | 3 | HIIT: After a 5-minute warm-up at 70% of peak heart rate (HRpeak) (determined by an initial incremental treadmill test), each participant performs 8 intervals of 1-minute vigorous walking at > 95% HRpeak, with 2-minute slow walking intervals in between for recovery.  MICT: The peak heart rate is set at 60%. | Body mass [kg] Fat mass [kg] Muscle mass [kg] Fat-free mass [kg] |
| Toohey 2016 | Australia | colon (n = 1), cervical (n = 1), melanoma (n = 1), ovarian (n = 2), breast (n=9) and a diagnosis breast and uterine (n=1) and breast and liver (n=1) | LVHIIT：8 CLMIT：8 | LVHIIT：8(100%) CLMIT：8(100%) | 51.6± 13.01 | LVHIIT：10 CLMIT：20 | 3 | 12 | HIIT group performs interval training (≥85% maximum heart rate).  MICT group performs continuous aerobic training (≤55% maximum heart rate). | Waist(cm) RHR(BPM) SBP(mmHg) DBP(mmHg) STS(5) (s) 6MWT(m) Mass(kg) Fat% Fat(kg) Lean(kg) |
| Toohey 2018 | Australia | Participant cancer diagnosis included 47 (82%) breast cancer, two (3%) ovarian cancer and one diagnosis of appendix, anal, cervical, liver, oesophageal, melanoma, leiomyosarcoma and unknown primary (15%). | LVHIIT：24 CLMIT：21 | LVHIIT：24(100%) CLMIT：21(100%) | LVHIIT：48±11.9 CLMIT：52 ±12.4 | LVHIIT：10 CLMIT：20 | 3 | 12 | HIIT group performs interval training (≥85% maximum heart rate).  MICT group performs continuous aerobic training (≤55% maximum heart rate). | Weight (kg) Body fat (%) Fat mass (kg) Lean mass (kg) Waist (cm) Hip (cm)1 Resting HR (bpm) STS (s) 6MWT (m) |
| Toohey 2020 | Australia | breast cancer survivors | HIIT:6 MICT:5 | HIIT:6(100%) MICT:5(100%) | HIIT:60±8.12 MICT:65±7.68 | 20-30 | 3 | 12 | HIIT: Increased to between 95 to 115 RPM.  MICT: 55-65% of maximum power. | Mean HR(b/min) |
