# Supplementary material for: Effects of high-intensity interval training (HIIT) versus moderate-intensity continuous training (MICT) on cardiopulmonary function, body composition, and physical function in cancer survivors: a meta-analysis of randomized controlled trials
Source: Front Physiol. 2025 Jun 13;16:1594574. doi: 10.3389/fphys.2025.1594574 (PMC12202225; doi:10.3389/fphys.2025.1594574)
Supplement: Supplementary file 3 [file Table3.docx]

***Cochrane ROB2***

**Table 1** assignment to intervention (the 'intention-to-treat' effect)

|  |  | **D1** | **D2** | **D3** | **D4** | **D5** | **Overall** |  |  |
| --- | --- | --- | --- | --- | --- | --- | --- | --- | --- |
| **1** | **bell 2021** | 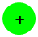 | 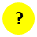 | 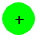 | 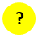 | 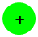 | 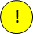 | 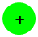 | **Low risk** |
| **2** | **devin 2015** | 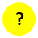 | 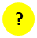 | 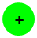 | 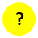 | 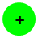 | 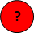 | 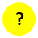 | **Some concerns** |
| **5** | **Isanejad 2023** | 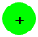 | 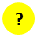 | 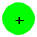 | 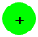 | 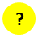 | 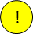 | 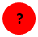 | **High risk** |
| **8** | **Northey 2018** | 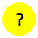 | 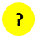 | 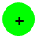 | 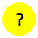 | 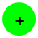 | 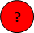 |  |  |
| **9** | **Schmitt 2016** | 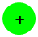 | 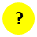 | 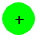 | 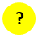 | 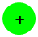 | 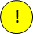 |  |  |
| **10** | **Toohey 2016** | 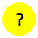 | 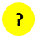 | 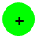 | 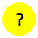 | 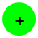 | 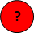 |  |  |
| **11** | **Toohey 2018** | 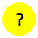 | 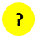 | 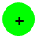 | 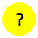 | 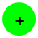 | 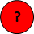 |  |  |
| **12** | **Toohey 2020** | 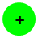 | 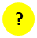 | 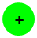 | 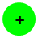 | 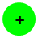 | 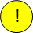 |  |  |

| **Table 2** adhering to intervention (the 'per-protocol' effect) | | | | | | | | | |
| --- | --- | --- | --- | --- | --- | --- | --- | --- | --- |
|  |  | **D1** | **D2** | **D3** | **D4** | **D5** | **D6** |  |  |
| **3** | **devin 2018** | 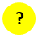 | 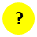 | 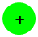 | 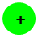 | 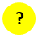 | 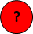 | 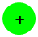 | **Low risk** |
| **4** | **dolan 2015** | 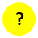 | 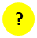 | 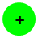 | 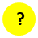 | 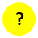 | 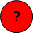 | 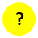 | **Some concerns** |
| **6** | **Moghadam 2021** | 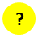 | 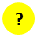 | 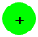 | 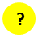 | 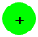 | 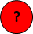 | 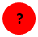 | **High risk** |
| **7** | **Moraitis 2023** | 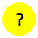 | 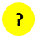 | 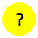 | 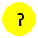 | 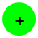 | 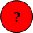 |  |  |
|  |  |  |  |  |  |  |  |  |  |

Domains:

D1: Bias arising from the randomization process.

D2: Bias due to deviations from intended intervention.

D3: Bias due to missing outcome data.

D4: Bias in measurement of the outcome.

D5: Bias in selection of the reported result.
